# Efficacy of letrozole combined with recombinant human growth hormone in boys aged ≥10 years with advanced bone age and impaired growth potential: a retrospective cohort study

**DOI:** 10.3389/fendo.2026.1940392

**Published:** 2026-09-24

**Authors:** Peipei Liu, Xiyan Lu, Miao Huang, Mingzhu Li, Wujin Wang, Guoqing Dong

**Affiliations:** Shenzhen Maternity and Child Healthcare Hospital, Women and Children’s Medical Center, Southern Medical University, Shenzhen, Guangdong, China

**Keywords:** adult height improvement, advanced bone age, growth hormone, letrozole, pubertal boys

## Abstract

**Introduction:**

In boys aged ≥10 years with advanced bone age and impaired growth potential (baseline height SDS < −1.5, BA-CA ≥1.5 years, and PAH < −2 SDS), growth hormone monotherapy, while promoting linear growth, fails to delay skeletal maturation, thus limiting therapeutic efficacy. Although letrozole combined with growth hormone may theoretically “decouple” height gain from epiphyseal closure, high-quality evidence specific to the advanced bone age population remains insufficient, and concerns regarding baseline bias and lack of long-term safety data persist. This study, based on real-world data, aims to clarify the benefits and risks of this combination regimen in promoting linear growth, delaying bone age progression, and improving adult height outcomes.

**Methods:**

A retrospective analysis was conducted on clinical data from 64 boys aged ≥10 years with advanced bone age and impaired growth potential treated in our hospital from June 2020 to December 2025 (baseline height SDS < −1.5, BA-CA ≥1.5 years, and PAH < −2 SDS). Patients were divided into a combination therapy group (letrozole + rhGH, n = 36) and a monotherapy group (rhGH alone, n = 28) according to treatment regimen. Changes in height, bone age, annual growth velocity, the ratio of bone age advancement to chronological age progression (ΔBA/ΔCA), and sex hormone levels were compared between the two groups before and after treatment.

**Results:**

The mean annual growth velocity in the combination group was (7.7 ± 1.2) cm/year, significantly higher than that in the monotherapy group [(6.7 ± 1.4) cm/year, *P* < 0.05]. The ΔBA/ΔCA ratio in the combination group was 0.85, significantly lower than that in the monotherapy group (1.12, *P* < 0.001), indicating better control of bone age progression. Among the 33 patients who had completed treatment, the mean final height was (171.2 ± 3.5) cm in the combination group, compared to (169.8 ± 4.1) cm in the monotherapy group (the difference was not statistically significant, P = 0.291).

**Discussion:**

In boys aged ≥10 years with advanced bone age and impaired growth potential, letrozole combined with rhGH significantly improved annual growth velocity and delayed bone age progression compared with rhGH monotherapy. However, the difference in final adult height among those who completed treatment was not statistically significant (1.4 cm, P = 0.291). The significantly better control of bone age advancement (ΔBA/ΔCA) and higher target-height attainment rate suggest a potential benefit of adding letrozole, but larger studies with more participants reaching final height are needed to confirm a meaningful effect on adult height.

## 1 Introduction

Rapidly progressive puberty (RPP) or advanced bone age with impaired growth potential in male children is a common concern in pediatric endocrinology clinics. In these patients, rapidly elevated sex hormone levels after pubertal onset accelerate epiphyseal maturation, leading to diminished growth potential and compromised final adult height (FAH) (1). Aromatase inhibitors (AIs), such as letrozole, may delay epiphyseal closure by inhibiting estrogen production, thereby offering a possibility to prolong the growth period (2). Recently, case reports and small-sample studies on the combination of rhGH and AIs in boys with advanced bone age and impaired growth potential have gradually increased; however, more clinical evidence is needed to confirm its definitive efficacy and impact on final height. This retrospective cohort study aimed to compare the efficacy of letrozole combined with rhGH versus rhGH monotherapy in treating boys aged ≥10 years with advanced bone age and impaired growth potential (defined as baseline height SDS < -1.5, BA-CA ≥ 1.5 years, and PAH < -2 SDS), providing evidence for optimizing clinical treatment strategies.

## 2 Subjects and methods

### 2.1 Study subjects

We retrospectively collected medical records of male patients who were first diagnosed with “advanced bone age with impaired growth potential during adolescence” and treated with either letrozole combined with rhGH or rhGH alone at the Department of Pediatric Endocrinology, Shenzhen Maternity and Child Healthcare Hospital from June 2020 to December 2025.

Inclusion criteria: ① Age ≥10 years; ②Clinical diagnosis of impaired growth potential, defined as height below the 3rd percentile or < -2 SDS or height between -1.5 SDS and -2.0 SDS but with a predicted adult height (PAH) < -2 SDS based on the Bayley-Pinneau method and a documented growth velocity decline (Δheight SDS < -1 over 1 year);all included patients had baseline height SDS < -1.5 and baseline PAH SDS < -2.0; the additional growth velocity decline was used to support clinical diagnosis but was not a mandatory inclusion criterion.③ Bone age (BA) exceeding chronological age (CA) by ≥1.5 years before treatment, assessed by the Greulich-Pyle (G-P) atlas method performed by a single senior pediatric endocrinologist (with intra-observer agreement > 95%);④ Completion of at least 6 months of standardized treatment with available follow-up records.

Exclusion criteria: ① Presence of severe chronic diseases, chromosomal abnormalities, intracranial tumors, or other endocrine disorders affecting growth (e.g. congenital adrenal hyperplasia, uncontrolled hypothyroidism);② growth hormone deficiency (GHD) was considered unlikely based on clinical evaluation (no extreme short stature, no documented growth velocity < 3 cm/year, and no delayed bone age), normal serum IGF-1 levels for age and sex, and appropriate pre-treatment growth velocity in all included patients. However, formal GH stimulation testing was not performed as part of the routine clinical workup, given that GHD is typically associated with delayed rather than advanced bone age — the defining feature of our study population. Consequently, while classical GHD was deemed unlikely, the absence of stimulation testing means that mild or partial GHD cannot be completely excluded; this limitation is acknowledged in the Discussion. ③ Severely incomplete data, defined as missing more than 20% of key follow-up parameters (height, bone age, or hormone levels) or absence of at least two consecutive height measurements during treatment; ④Poor treatment adherence, defined as less than 80% of prescribed doses taken based on medication refill records and patient diaries, or voluntary switching of the regimen during treatment.

Pubertal staging by Tanner criteria was not systematically recorded in all cases, therefore, pubertal status was inferred from baseline hormone levels and bone age advancement, which suggested that most patients were in mid-to-late puberty. This limitation is acknowledged in the Discussion.

This retrospective study was approved by the Institutional Review Board of Shenzhen Maternity and Child Healthcare Hospital (No. 092). The IRB waived the requirement for written informed consent for this retrospective chart review study, as all data were de-identified and the study involved no more than minimal risk to subjects. The study was conducted in accordance with the principles of the Declaration of Helsinki. Although the baseline height SDS of some patients did not reach the traditional threshold of -2 SD, the combination of advanced bone age, declining growth velocity, and compromised predicted adult height constituted a clear clinical indication for intervention, and all treatments were prescribed after thorough discussion with families. The ethics committee specifically reviewed and approved this pragmatic approach.

### 2.2 Study methods

#### 2.2.1 Grouping

Patients were divided into two groups according to treatment regimen: combination therapy group (letrozole tablet 2.5 mg/d orally + recombinant human growth hormone (rhGH, 0.1-0.2 IU/kg/d) and monotherapy group (rhGH alone at the same dose).

#### 2.2.2 Outcome measures

We collected patients’ height, weight, bone age (assessed by the same senior physician using the Greulich-Pyle atlas method, with repeated measurements to ensure consistency), and sex hormone levels (testosterone, estradiol, LH, and FSH were measured by electrochemiluminescence immunoassay (ECLIA) using a Cobas e601 analyzer, Roche Diagnostics) before treatment and at the last follow-up. Annual growth velocity (GV, cm/year) and the ratio of bone age advancement to chronological age progression (ΔBA/ΔCA) were calculated. Height standard deviation scores (HtSDS) were calculated based on the same Chinese reference data at baseline and at the end of treatment or at final height.

#### 2.2.3 Treatment discontinuation criteria

Discontinuation of rhGH and letrozole followed the following clinical criteria:

1. Growth velocity < 1.5 cm/year and bone age ≥ 16 years (near epiphyseal closure) – this was considered as achieving near-final adult height; final adult height (FAH) was defined as the measured height at the last visit when growth velocity dropped below 1.5 cm/year and bone age reached ≥ 16.5 years for boys, or when the patient and family decided to stop; target height (THt) was calculated from mid-parental height using the formula: (father’s height + mother’s height + 13)/2 for boys, and the attainment rate was calculated as (FAH/THt) × 100%;
2. Bone age reaching 16.5-17 years, regardless of height satisfaction;
3. Occurrence of intolerable adverse drug reactions (e.g.psychological effects, severe arthralgia, or liver function abnormalities);
4. Voluntary request by the patient or parents to terminate treatment. Discontinuation criteria were applied consistently in both groups.

#### 2.2.4 Statistical analysis

Data were processed using SPSS 26.0 software. Continuous data were expressed as mean ± standard deviation (x±s) and compared between groups using independent samples t-test. All tests were two-sided, and a P < 0.05 was considered statistically significant. Categorical data were expressed as n (%) and compared using the χ² test or Fisher’s exact test when any expected cell count was < 5. To adjust for potential baseline confounders, multiple linear regression analysis was performed with height gain and ΔBA/ΔCA as dependent variables, and treatment group (combination vs. monotherapy), age, baseline height SDS, baseline BA-CA difference, baseline PAH SDS, and baseline testosterone as independent variables.

## 3 Results

### 3.1 Comparison of baseline characteristics

A total of 64 patients were included, with 36 in the combination group and 28 in the monotherapy group. There were no statistically significant differences between the two groups in pretreatment age, height, height standard deviation score, bone age, body mass index (BMI), or levels of testosterone, LH, and FSH (P > 0.05), indicating comparability (**Table 1**).

**Table 1:** Baseline characteristics of the two groups.

Variable	Combination group (n=36)	Monotherapy group (n=28)	*P*
Age (years)	12.2 ± 1.3	12.1 ± 1.5	0.776
Height (cm)	155.2 ± 6.3	154.1 ± 7.0	0.506
Bone age (years)	14.1 ± 1.2	13.9 ± 1.4	0.519
Height SDS	-1.6 ± 0.8	-1.7 ± 0.9	0.603
BA-CA difference (years)	1.9 ± 0.4	1.8 ± 0.5	0.366
Baseline PAH SDS	-2.2 ± 0.5	-2.0 ± 0.6	0.151
Baseline target height (THt, cm)	172.5 ± 4.2	171.8 ± 4.5	0.525
Baseline GV decline (ΔHtSDS over 1 yr)	-1.3 ± 0.4	-1.1 ± 0.5	0.089
Body mass index (kg/m²)	19.1 ± 3.2	18.7 ± 3.5	0.634
Testosterone (μg/L)	1.52 ± 0.89	1.48 ± 0.92	0.886
LH(IU/L)	2.91 ± 1.85	2.56 ± 1.63	0.428
FSH(IU/L)	4.68 ± 3.02	4.33 ± 2.86	0.640

### 3.2 Changes in growth parameters

The mean treatment duration was 1.5 ± 0.7 years in the combination group and 1.3 ± 0.6 years in the monotherapy group, with no statistically significant difference (P > 0.05). The mean height gain and annual growth velocity during treatment were significantly higher in the combination group than in the monotherapy group (P < 0.05). Moreover, the combination group showed better control of bone age progression (**Table 2**).

**Table 2:** Comparison of growth parameters after treatment between the two groups.

Group	n	Height gain (cm)	Annual growth velocity (cm/year)	ΔBA/ΔCA
Combination group	36	11.7 ± 3.1	7.7 ± 1.2	0.85 ± 0.15
Monotherapy group	28	9.4 ± 3.5	6.7 ± 1.4	1.12 ± 0.21
*t*		2.84	3.06	-6.02
*P*		0.006	0.003	<0.001

### 3.3 Bone age control effect

The ΔBA/ΔCA ratio in the combination group was significantly lower than that in the monotherapy group *(P* < 0.001), indicating that under combination therapy, bone age advanced more slowly than chronological age, effectively prolonging the growth window (**Table 2**).

### 3.4 Final height outcomes

By the data cutoff, a total of 33 patients (18 in the combination group, 15 in the monotherapy group) had completed treatment. The mean final height in the combination group was (171.2 ± 3.5) cm, higher than that in the monotherapy group [(169.8 ± 4.1) cm], but the difference did not reach statistical significance (P = 0.291). The higher target height attainment rate (though not statistically significant) based on mid-parental height was (98.5 ± 3.1)% in the combination group and (96.2 ± 4.0)% in the monotherapy group (P = 0.078). The proportion of patients reaching or exceeding target height was numerically higher in the combination group (83.3% vs. 60.0%), although the difference was not statistically significant (P = 0.139) (**Table 3**).Final height SDS values were -0.8 ± 0.6 in the combination group and -1.1 ± 0.7 in the monotherapy group (P = 0.182).

**Table 3:** Comparison of growth parameters after treatment completion between the two groups.

Variable	Combination group (n=18)	Monotherapy group (n=15)	*P*
Treatment duration (years)	1.5 ± 0.7	1.3 ± 0.6	0.38
Final height (cm)	171.2 ± 3.5	169.8 ± 4.1	0.291
Final height SDS	-0.8 ± 0.6	-1.1 ± 0.7	0.182
Target height (THt) attainment rate (%)	98.5 ± 3.1	96.2 ± 4.0	0.078
Achieving target height, n (%)	15 (83.3%)	9 (60.0%)	0.139

### 3.5 Adjusted analysis for baseline confounders

To address potential indication bias due to the non-randomized design, we performed multiple linear regression analysis. After adjustment, combination therapy remained significantly associated with greater height gain (β = 2.1, 95% CI: 0.8 to 3.4, P = 0.002) and lower ΔBA/ΔCA (β = -0.25, 95% CI: -0.38 to -0.12, P < 0.001).These associations should be interpreted with caution, as residual confounding from unmeasured variables (e.g.socioeconomic status, dietary factors, adherence nuances) cannot be excluded given the non-randomized, retrospective design.

### 3.6 Adverse events between the two groups

In the combination group, 18 out of 36 patients (50.0%) reported at least one adverse event, compared to 9 out of 28 patients (32.1%) in the monotherapy group (P = 0.203). Common adverse events in the combination group included transient hand tremor (5 cases, 13.9%), hyperuricemia (uric acid >420 μmol/L, 6 cases, 16.7%), and androgen-related manifestations (acne, irritability, etc., 4 cases, 11.1%). In the monotherapy group, the main adverse events were borderline elevated blood glucose (4 cases, 14.3%) and mild thyroid function abnormalities (3 cases, 10.7%). All adverse events were mild, requiring neither hospitalization nor permanent discontinuation. Two patients in the combination group temporarily discontinued treatment due to hand tremor under medical guidance, with symptom relief and subsequent treatment resumption. No serious adverse events occurred in either group (**Table 4**).

**Table 4:** Adverse events after treatment in the two groups.

Variable	Combination group (n=36)	Monotherapy group (n=28)	*P*
Any adverse event	18 (50.0)	9 (32.1)	0.203
Serious adverse event	0 (0)	0 (0)	–
Discontinuation due to adverse event	2 (5.6)	0 (0)	0.501
Hand tremor	5 (13.9)	0 (0)	0.063
Androgen-related manifestations	4 (11.1)	0(0)	0.123
Hyperuricemia	6 (16.7)	1 (3.6)	0.130
Elevated blood glucose	2 (5.6)	4 (14.3)	0.396
Thyroid function abnormality	1 (2.8)	3 (10.7)	0.315

Serious adverse events refer to events leading to hospitalization, life-threatening conditions, or permanent disability; none were observed in this study.

## 4 Discussion

Advanced bone age with impaired growth potential in boys aged ≥10 years is a common growth and development concern in clinical practice. In these patients, rapidly elevated sex hormone levels after pubertal onset, particularly estrogen which plays a key role in epiphyseal closure, accelerate bone age progression, leading to significantly shortened growth potential and compromised final adult height (FAH) (3). According to statistics, approximately 5%-20% of adolescents experience varying degrees of growth retardation, and patients with advanced bone age face greater treatment difficulty due to a higher risk of premature epiphyseal closure (4). Current treatment options for boys with advanced bone age and impaired growth potential include gonadotropin-releasing hormone analogs (GnRHa), growth hormone (GH) supplementation, and aromatase inhibitors. However, these methods have limitations such as unstable efficacy and potential side effects. GnRHa is effective in delaying bone age progression and sexual development, but some patients experience limited height gain (5). Growth hormone monotherapy promotes height increase but does not delay bone age progression (6). Aromatase inhibitors such as letrozole have shown advantages in inhibiting estrogen synthesis and delaying bone age progression, attracting attention especially in the regulation of abnormally accelerated puberty in males (7). A randomized controlled trial also demonstrated that letrozole treatment in boys with delayed puberty does not negatively affect bone metabolism in the short term, highlighting the regulatory role of letrozole in pubertal sex hormone dynamics (9). Nevertheless, single-agent therapy often fails to balance bone age control and height gain simultaneously, and achieving this balance remains a clinical challenge.

In recent years, case reports and small-sample studies on the combination of rhGH and AIs in boys with advanced bone age and impaired growth potential have gradually increased. A study on East Asian adolescent males with idiopathic short stature (ISS) found that rhGH combined with AIs effectively improved growth velocity and controlled bone age progression, thereby increasing final adult height; however, the optimal dose, timing, and safety of the combination regimen require further validation (10). Through a retrospective cohort analysis, our study specifically evaluated the efficacy and safety of letrozole combined with rhGH versus rhGH monotherapy in boys aged ≥10 years with advanced bone age and impaired growth potential. The novelty of our study lies in its focus on the challenging clinical subgroup of “advanced bone age,” in which the growth window is significantly shortened due to rapid pubertal progression, and conventional rhGH treatment often yields unsatisfactory results. Previous studies have mainly focused on the application of AIs in idiopathic short stature or growth hormone deficiency (11),evidence for the effectiveness of combination therapy in boys who have already entered puberty with significantly advanced bone age remains relatively limited. Our study found that combination therapy not only significantly increased annual growth velocity but, more importantly, resulted in a significantly lower ratio of bone age advancement to chronological age progression compared to the monotherapy group. This finding echoes reports by Giannopoulou et al. that letrozole treatment in patients with aromatase excess syndrome can delay bone age progression and improve final height (8). A real-world study in male patients in mid-puberty with reduced predicted adult height due to advanced bone age showed that AIs alone or combined with GH significantly slowed bone age progression and improved final adult height (12). Torres-Santiago investigated four treatment regimens (rhGH alone, GnRHa + rhGH, AIs alone, and AIs + rhGH) in pubertal boys with ISS and advanced bone age, and found that the combination of AIs and rhGH helped boys smoothly navigate puberty, selectively reduced estrogen levels, and more effectively improved adult height (2). Furthermore, AIs have shown potential in improving height outcomes in children with bone age advancement due to other conditions. In children with congenital adrenal hyperplasia and advanced bone age, anastrozole treatment effectively slowed skeletal maturation, significantly improving predicted adult height and bone-age-adjusted height, providing further evidence from another pathological perspective that AIs are effective in controlling bone age acceleration and gaining growth time (13). The combined application of letrozole during the pubertal growth spurt effectively “decouples” the rates of height gain and bone age maturation, providing key data supporting the extension of effective growth period.

Brief comparison with alternative strategies: While GnRHa combined with GH is an alternative approach for delaying bone age progression, it suppresses the pituitary-gonadal axis and may blunt the pubertal growth spurt (5).AIs, by contrast, selectively block estrogen synthesis without suppressing gonadotropins or testosterone, potentially offering a more favorable balance between growth promotion and bone age control. Head-to-head comparisons between AI+GH and GnRHa+GH are lacking and represent an important direction for future research (2, 11).

Our retrospective cohort analysis found that the observed adverse events during treatment were generally mild and transient. However, we acknowledge that this study did not systematically evaluate long-term safety parameters including bone mineral density (BMD), lipid profiles, insulin resistance (HOMA-IR), or testicular volume. These parameters are particularly relevant given that letrozole suppresses estrogen synthesis, which may have implications for bone health, cardiovascular risk, and gonadal function during the critical pubertal period. Therefore, while the short-term adverse event profile appeared acceptable in this limited sample with short follow-up, long-term safety cannot be inferred from this study and requires dedicated prospective evaluation with systematic monitoring. Transient adverse events such as hand tremor were consistent with mild adverse reactions reported for aromatase inhibitors in other clinical studies (14, 15). These side effects may be related to hormonal fluctuations and the drug’s impact on the central nervous system; they did not lead to permanent treatment discontinuation in this short-term study. The low incidence of dose-dependent side effects in this short-term observation does not permit conclusions about the safety margin of the current dosage or superiority over other hormonal therapies. Direct comparisons of safety profiles between different treatment regimens were not possible in this study and require dedicated comparative trials. In future clinical practice, attention should be paid to monitoring and managing side effects, with timely adjustment of treatment regimens to improve patient adherence and treatment continuity.

Naturally, this study has several limitations. First, as a single-center retrospective study, the sample size is relatively limited, and treatment assignment was non-randomized, which may introduce selection bias. Although baseline characteristics were comparable between the two groups, confounding factors due to physician clinical decisions or parental preferences cannot be completely excluded (16). The adjusted analyses should be interpreted as demonstrating associations rather than establishing causal treatment effects, particularly because these analyses were performed for height gain and ΔBA/ΔCA rather than for final adult height. Residual confounding from unmeasured variables (e.g., socioeconomic status, dietary factors, adherence nuances) may also exist. Second, the inclusion of patients with baseline height SDS around -1.6, which is above the classic -2 SD threshold for short stature, may raise questions about the indication for treatment. However, as mentioned, all included boys had a combination of advanced bone age, declining growth velocity, and a predicted adult height well below their genetic potential (PAH < -2 SDS), which constitutes a clinically meaningful growth impairment. The conclusions of this study are therefore most applicable to the specific subgroup of boys aged ≥10 years with borderline short stature (height SDS between -1.5 and -2.0) but significantly impaired growth potential due to advanced bone age and declining growth velocity, rather than to all short boys with advanced bone age. Third, although bone age assessments were performed by the same senior physician using the Greulich-Pyle atlas method, some subjective bias remains. We did not use automated software or ultrasound-based methods, which could improve objectivity (17). Fourth, follow-up durations were not uniform, and only a subset of patients (33 cases) had reached final height. The 1.4 cm difference in final height did not reach statistical significance in this underpowered sample. The higher target height attainment rate in the combination group (though not statistically significant) (98.5% vs. 96.2%, P = 0.078) provides suggestive evidence of a potential benefit, but this finding requires confirmation in adequately powered randomized trials. Fifth, we did not systematically collect Tanner staging data, which limits our ability to precisely characterize pubertal status; although baseline hormone levels and bone age advancement provided indirect evidence of mid-pubertal status, this remains an important limitation. Future studies should incorporate detailed pubertal staging. Sixth, formal GH stimulation testing was not performed to exclude GHD, as this was not part of the routine clinical evaluation for boys with advanced bone age, in whom GHD is clinically atypical. Instead, GHD was excluded based on clinical assessment, normal IGF-1 levels, and appropriate pre-treatment growth velocity. However, the absence of stimulation testing means that mild or partial GHD cannot be completely ruled out. While this does not undermine the primary findings-since advanced bone age is not a feature of GHD and the study population was specifically defined by this characteristic-it should be acknowledged as a methodological limitation. Future prospective studies incorporating GH stimulation tests or GH-related biomarkers could further clarify whether the observed treatment effects are influenced by underlying GH status. In the future, multicenter, randomized controlled prospective studies will more reliably validate the conclusions of this study and further explore optimal drug dosage, treatment initiation timing, duration, and biomarkers predictive of treatment response.

Finally, The lack of statistical significance in final height difference (1.4 cm, P = 0.291) should be interpreted cautiously. The study was not powered to detect a small difference in final height, and the numerical advantage in the combination group, along with numerically better target-height attainment rates, suggests a potential clinical benefit that warrants further investigation. We have revised our conclusion to reflect this nuance.

In conclusion, for boys aged ≥10 years with advanced bone age and impaired growth potential, the combination of letrozole and rhGH effectively promoted linear growth and delayed bone-age progression during treatment. While the final adult height difference was not statistically significant in this limited sample, the improved target-height attainment rate and bone-age control indicate a promising role for this regimen. Given the marginal clinical effect and the need for confirmatory evidence, we recommend that the decision to add letrozole be individualized, and further prospective, adequately powered studies are required to establish its efficacy on final adult height. These findings provide insights for managing this specific clinical subgroup; however, the results should not be generalized to all boys with short stature. Future studies should focus on efficacy differences across different bone age stages and long-term safety.

## Data Availability

The raw data supporting the conclusions of this article will be made available by the authors, without undue reservation.
